# Minocycline Counteracts Ectopic Calcification in a Murine Model of Pseudoxanthoma Elasticum: A Proof-of-Concept Study

**DOI:** 10.3390/ijms23031838

**Published:** 2022-02-06

**Authors:** Elise Bouderlique, Lukas Nollet, Emmanuel Letavernier, Olivier M. Vanakker

**Affiliations:** 1 UMR S 1155, Institut National de la Santé et de la Recherche Médicale (INSERM), Sorbonne Université, 75020 Paris, France; elise.bouderlique@inserm.fr (E.B.); emmanuel.letavernier@aphp.fr (E.L.); 2 Center for Medical Genetics, Ghent University Hospital, 9000 Ghent, Belgium; lukas.nollet@ugent.be; 3 Department of Biomolecular Medicine, Ghent University, 9000 Ghent, Belgium; 4 Ectopic Mineralization Research Group, 9000 Ghent, Belgium

**Keywords:** ectopic calcification, pseudoxanthoma elasticum, minocycline, DNA damage response, PARP1, genetic disorders, treatment

## Abstract

Pseudoxanthoma elasticum (PXE) is an intractable Mendelian disease characterized by ectopic calcification in skin, eyes and blood vessels. Recently, increased activation of the DNA damage response (DDR) was shown to be involved in PXE pathogenesis, while the DDR/PARP1 inhibitor minocycline was found to attenuate aberrant mineralization in PXE cells and zebrafish. In this proof-of-concept study, we evaluated the anticalcifying properties of minocycline in *Abcc6^−/−^* mice, an established mammalian PXE model. *Abcc6^−/−^* mice received oral minocycline supplementation (40 mg/kg/day) from 12 to 36 weeks of age and were compared to untreated *Abcc6^−/−^* and *Abcc6^+/+^* siblings. Ectopic calcification was evaluated using X-ray microtomography with three-dimensional reconstruction of calcium deposits in muzzle skin and Yasue’s calcium staining. Immunohistochemistry for the key DDR marker H2AX was also performed. Following minocycline treatment, ectopic calcification in *Abcc6^−/−^* mice was significantly reduced (−43.4%, *p* < 0.0001) compared to untreated *Abcc6^−/−^* littermates. H2AX immunostaining revealed activation of the DDR at sites of aberrant mineralization in untreated *Abcc6^−/−^* animals. In conclusion, we validated the anticalcifying effect of minocycline in *Abcc6^−/−^* mice for the first time. Considering its favorable safety profile in humans and low cost as a generic drug, minocycline may be a promising therapeutic compound for PXE patients.

## 1. Introduction

Ectopic calcification is characterized by an abnormal deposition of calcium hydroxyapatite crystals in soft tissues including skin, eyes and blood vessels [1]. Although the precise pathophysiology of ectopic calcification has currently not been fully elucidated, it is believed to result from a chronic imbalance between procalcifying factors (calcium, phosphorus, vitamin D) and anticalcifying factors (inorganic pyrophosphate, fetuin-A, matrix gla protein) [2]. Apart from circulating ions and proteins forming the ‘systemic’ component of ectopic calcification pathogenesis, ‘local’ factors such as the presence of mesenchymal cells, which transdifferentiate towards an osteogenic phenotype, further contribute to soft tissue mineralization [3,4]. Ectopic calcification is a hallmark of some of the most frequent Western disorders such as diabetes mellitus, chronic kidney disease (CKD) and calcific aortic valve stenosis while also being abundantly present in aging individuals (>70 years) [5]. In these cases, aberrant soft tissue mineralization (e.g., vascular calcification) causes significant morbidity due to organ function impairment (e.g., heart failure resulting from increased systemic vascular resistance) as well as increased mortality due to ischemia-related complications (e.g., stroke, myocardial infarction) [6,7]. As no effective treatment options for ectopic calcification in humans currently exist, basic and translational research into its pathogenesis remains of utmost importance. 

Studies in rare Mendelian diseases have been instrumental in understanding the multitude of cellular signaling pathways involved in the ectopic calcification process. Pseudoxanthoma elasticum (PXE; OMIM #264800) is considered a paradigm of hereditary ectopic calcification disorders and is typically caused by bi-allelic pathogenic variants in the *ABCC6* gene [8]. *ABCC6* encodes a transmembrane ATP-dependent efflux transporter primarily expressed on the basolateral membrane of hepatocytes and, to a lesser extent, in renal proximal tubular cells, transferring a still-unknown substrate [9,10]. Clinically, PXE patients develop extensive mineralization of elastic fibers from late childhood onwards, affecting the mid dermis of the skin (resulting in yellowish papular lesions and excessive skin folds), the retinal Bruch membrane (complicated by choroidal neovascularization, hemorrhage and vision loss) and the medial layer of arteries (predisposing to stroke and peripheral artery disease) [11]. To date, no effective therapies significantly halting the progression of ectopic calcification are available for PXE patients, conferring a high unmet medical need to this patient population.

We recently showed that excessive and sustained activation of the DNA damage response (DDR) and poly-ADP ribose polymerase 1 (PARP1) signaling is a major characteristic of the ‘local’ component of PXE pathophysiology, and that targeted inhibition of PARP1 by minocycline significantly attenuates ectopic calcification in PXE cells and tissues [12]. Mechanistically, the DDR consists of a signaling cascade that is initiated following DNA strand breaks and includes both sensing proteins such as H2AX, ATM and PARP1, as well as effector proteins such as p21 and p53, resulting in cell cycle arrest and ultimately either DNA break repair or apoptosis/senescence of the cell [13]. Additionally, PARP1 downstream signaling includes the transcription factors STAT1/3, which in turn activate the epigenetic modifier TET1, which is involved in osteochondrogenic transdifferentiation of the cell, and RUNX2, a master regulator of ectopic calcification [14,15]. Interestingly, PARP enzymes perform a specific post-translational modification known as PARylation, in which PAR moieties are attached to target proteins. In the context of ectopic calcification, it has been shown that PARylation of extracellular matrix proteins such as fibronectin and annexins increases their ability to actively bind calcium ions through the pyrophosphate groups present in PAR, hence forming a nidus on which calcium crystallization can occur [16]. In PXE, we previously showed that this PARylated protein fraction is significantly increased both in vitro and in vivo compared to healthy controls [12].

Overactivation of the DDR and PARP1 signaling pathways has also been demonstrated in other ectopic calcification disorders such as diabetes mellitus and CKD, suggesting that it is a universal and common disease mechanism in aberrant tissue mineralization [16,17]. Inhibition of the DDR by treatment with PARP1 inhibitors such as minocycline was shown to significantly ameliorate the ectopic calcification phenotype in experimental models of diabetes mellitus and CKD [16,18]. In PXE, we demonstrated similar treatment effects in vitro (PXE patient-derived dermal fibroblasts) as well as in vivo in CRISPR-Cas9-engineered *Abcc6^−/−^* zebrafish, a validated vertebrate PXE animal model, showing an average reduction in ectopic calcification of 60% [12].

However, to increase the translational potential of minocycline for treatment of human PXE patients, validation of its anticalcifying effects in *Abcc6*-deficient mice, considered the gold standard mammalian PXE model, was first warranted. In this study, we therefore investigated whether oral minocycline supplementation could attenuate ectopic calcification in *Abcc6^−/−^* mice using vibrissae dermal sheath mineralization as a read-out. We conclude that minocycline treatment significantly reduces ectopic calcification in PXE mice (>40% reduction in vibrissae mineralization), hence providing evidence that minocycline may potentially be effective in treating ectopic calcification in patients with PXE. 

## 2. Results

### 2.1. Oral Minocycline Treatment Significantly Reduces Ectopic Calcification in Abcc6^−/−^ Mice

**Figure 1:**
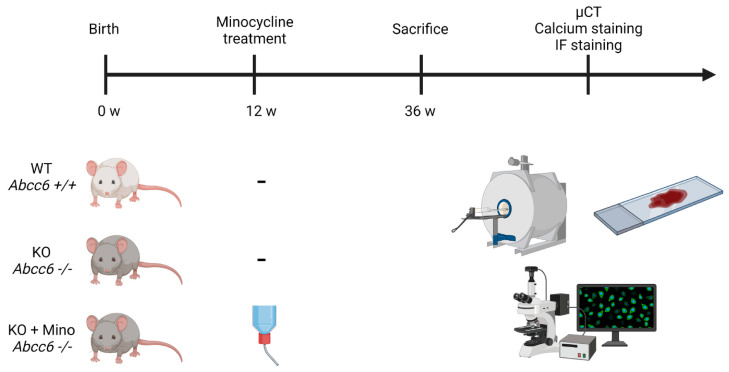
Study design. Treatment group *Abcc6^−/−^* mice received oral minocycline supplementation from 12 weeks to 36 weeks of age (KO + Mino), while control group *Abcc6^−/−^* (KO) and *Abcc6^+/+^* (WT) siblings received drinking water without minocycline supplementation. MicroCT imaging, Yasue’s calcium staining and immunofluorescence staining were performed after euthanasia. µCT = micro computed tomography; IF = immunofluorescence; KO = knock-out; mino = minocycline; w = weeks; WT = wild type.

*Abcc6*-deficient mice are known to fully recapitulate the histopathological and ultrastructural features of human PXE, as evidenced by late-onset, widespread and progressive ectopic calcification in soft tissues [19]. Aberrant mineralization of the connective tissue sheath surrounding the hair follicles of vibrissae is typically observed in *Abcc6^−/−^* mice and is considered a robust read-out and reliable biomarker of the ectopic calcification phenotype in PXE [20]. In this study, female *Abcc6^−/−^* mice were treated with oral minocycline supplementation in drinking water at a dose of 40 mg/kg/day (Human Equivalent Dose: 3.3 mg/kg/day; corresponding to the FDA-approved maximal daily dose of 200 mg in humans) from 12 to 36 weeks of age (treatment group: *n* = 6) (Figure 1). *Abcc6^−/−^* (*n* = 6) and *Abcc6^+/+^* (*n* = 6) littermates received normal drinking water without minocycline supplementation (control groups).

**Figure 2:**
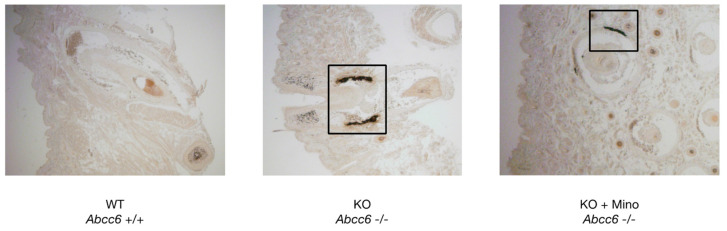
Yasue’s calcium staining of paraffin-embedded muzzle skin. Ectopic calcification in the connective tissue sheath of vibrissae is absent in *Abcc6^+/+^* mice (left panel), while extensive calcium crystal deposits are present in *Abcc6^−/−^* littermates (middle panel). Minocycline treatment markedly reduced vibrissae sheath calcification in *Abcc6^−/−^* mice (right panel). Representative images from all groups are shown. KO = knock-out; mino = minocycline; WT = wild type.

After euthanasia, histopathologic examination of paraffin-embedded muzzle skin from control *Abcc6^−/−^* mice revealed extensive calcification of the vibrissae sheaths as evidenced by large brown-black deposits on Yasue’s calcium staining (Figure 2; middle panel). By contrast, vibrissae sheath calcification was absent in *Abcc6^+/+^* mice (Figure 2; left panel). Following minocycline treatment of *Abcc6^−/−^* animals, calcification deposits were markedly decreased in size as evidenced by Yasue’s staining (Figure 2; right panel).

**Figure 3:**
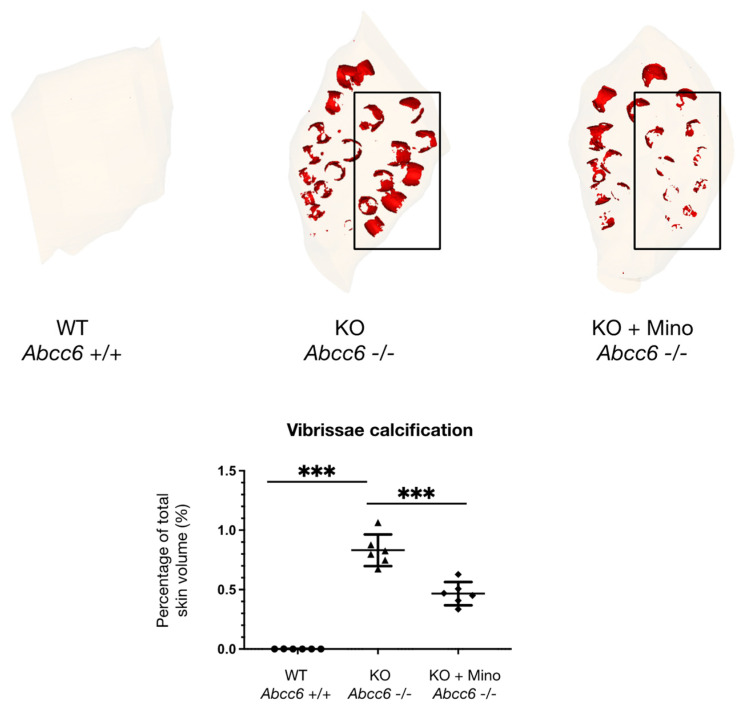
X-ray microtomography reconstruction of muzzle skin. µCT imaging and 3D reconstruction of paraffin-embedded muzzle skin revealed absent vibrissae calcification in *Abcc6^+/+^* mice (*n* = 6), widespread calcification in untreated *Abcc6^−/−^* littermates (*n* = 6) and markedly attenuated calcification in minocycline treated *Abcc6^−/−^* animals (*n* = 6). Quantitative analysis of calcification volume showed a significant reduction in vibrissae sheath mineralization following minocycline treatment. KO = knock-out; mino = minocycline; WT = wild type; *** *p* < 0.0001.

To enable quantitative analysis of ectopic calcification burden in the animals and to evaluate the effect size of minocycline treatment, contralateral muzzle skin biopsies were embedded in paraffin and imaged using X-ray microtomography. Subsequently, three-dimensional reconstruction of calcified vibrissae sheaths was performed, followed by volume measurement of calcified areas normalized to total muzzle skin volume. As shown in Figure 3 and Supplemental Videos S1–S3, µCT reconstruction showed almost absent vibrissae calcification in *Abcc6^+/+^* mice (percentage calcified volume/total skin volume: 0.00051 ± 0.00078), whereas widespread calcification was observed in *Abcc6^−/−^* siblings (0.83 ± 0.13; *p* < 0.0001). Oral minocycline supplementation greatly reduced vibrissae sheath calcification in *Abcc6^−/−^* mice, with quantitative analysis revealing a significant 43.4% reduction in calcification volume compared to untreated *Abcc6^−/−^* animals (percentage calcified volume/total skin volume: 0.47 ± 0.10 vs. 0.83 ± 0.13; *p* < 0.0001).

### 2.2. Activation of the DNA Damage Response Colocalizes with Ectopic Calcification in Abcc6-Deficient Mice

**Figure 4:**
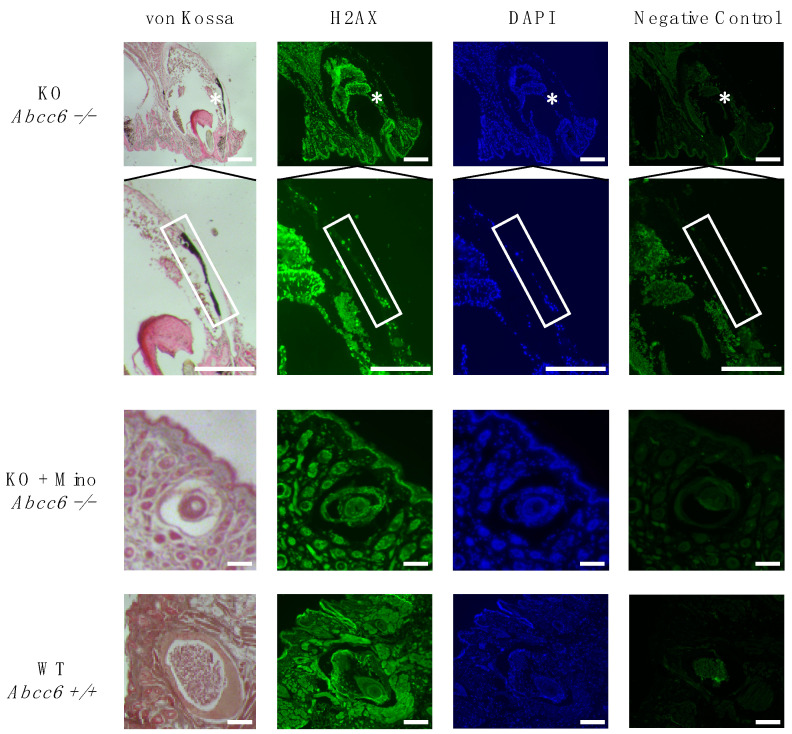
Immunofluorescence staining for the DDR marker H2AX in muzzle skin. Strongly positive staining for H2AX was observed in the nuclei of vibrissae sheath cells in untreated *Abcc6^−/−^* mice, colocalizing with areas of ectopic calcification as seen using von Kossa calcium staining, compared to minimal immunostaining and absent calcification in minocycline-treated *Abcc6^−/−^* and control *Abcc6^+/+^* animals. Scale bar = 200 µm. Areas marked with asterisks are additionally enlarged. DAPI = 4′,6-diamidino-2-phenylindole; H2AX = H2A histone family member X; KO = knock-out; mino = minocycline; WT = wild type.

As our group recently demonstrated that increased DDR activation is involved in the pathogenesis of ectopic calcification in PXE patient-derived dermal fibroblasts and lesional skin tissue as well as in a vertebrate PXE animal model (*Danio rerio*) [12], we evaluated whether a similar mechanism is also present in *Abcc6^−/−^* mice. To this end, immunofluorescence staining for the key DDR marker H2A histone family member X (H2AX) was performed on paraffin sections from muzzle skin. Strongly positive staining for H2AX was observed in the nuclei of vibrissae sheath cells in untreated *Abcc6^−/−^* mice, colocalizing with areas of extensive ectopic calcification as seen using von Kossa calcium staining, compared to minimal immunostaining and absent calcification in minocycline-treated *Abcc6^−/−^* and control *Abcc6^+/+^* animals (Figure 4).

## 3. Discussion

Ectopic calcification is a frequently encountered pathomechanism in common disorders such as diabetes mellitus, CKD and premature ageing. Although it results in substantial morbidity and mortality, no effective treatment options capable of halting the progression of ectopic calcification or reversing already existing calcium crystal deposits in humans exist to date. In recent work by our group, we showed that increased and sustained activation of the DDR and PARP1 signaling pathways contributes to ectopic calcification in the rare Mendelian disorder PXE, and that DDR inhibition by the PARP1 inhibitor minocycline could significantly counteract soft tissue mineralization in PXE cells and our *abcc6^−/−^* zebrafish model [12].

In the current study, we evaluated whether minocycline treatment could also attenuate the ectopic calcification phenotype in the main mammalian PXE animal model, that is, *Abcc6*-deficient mice.

Our main finding is that oral minocycline supplementation in *Abcc6^−/−^* mice at a dose of 40 mg/kg/day—corresponding to the FDA-approved maximal daily dose of 200 mg in humans—from 12 to 36 weeks of age resulted in a significant 43% reduction in ectopic calcification compared to untreated littermates. This treatment effect of minocycline was evaluated both qualitatively by means of Yasue’s calcium staining of muzzle skin and quantitatively by means of 3D reconstruction following X-ray microtomography. Hence, we can reliably conclude that minocycline is a potent anticalcifying compound in PXE mice.

Deposition of calcium hydroxyapatite crystals in the connective tissue sheath surrounding vibrissae hair follicles, starting as early as 5 weeks of age, is widely considered to be the main readout of ectopic calcification burden in *Abcc6^−/−^* mice and has been extensively validated in previous studies [20,21]. However, apart from vibrissae sheath calcification, *Abcc6*-deficient mice also present progressive mineralization in the retinal Bruch membrane, arterial blood vessels and renal papillae, though only at significantly older age (>12 months) and with substantial intra- and intersubject variability [19,22,23]. Therefore, future studies should perform longer treatments (>1 year) in larger groups of *Abcc6^−/−^* mice to evaluate a potential beneficial effect of minocycline at these alternative sites of ectopic calcification, which was beyond the scope and time limit of our current proof-of-concept study.

Additionally, we showed that activation of the DDR, as evidenced by positive immunostaining for the key DDR marker H2AX, colocalizes with ectopic calcification in vibrissae of *Abcc6*-deficient mice, while only minimal immunostaining was observed in minocycline-treated *Abcc6^−/−^* siblings. These findings are in line with recent results from our group in PXE patient-derived fibroblasts and *abcc6^−/−^* zebrafish, as well as with previous in vitro and in vivo studies in acquired ectopic calcification disorders such as CKD [12,16].

Minocycline is a semi-synthetic tetracycline with antibiotic properties against both Gram-positive and Gram-negative bacteria, as it interferes with the bacterial 30S ribosomal subunit, hence inhibiting protein synthesis [24]. To date, minocycline is mostly used in clinical practice for the treatment of acne vulgaris and sexually transmitted diseases. However, minocycline has been shown to have multiple nonbacteriostatic biological effects, including immunomodulation, prevention of apoptosis and inhibition of PARP1 [24]. As previously demonstrated by our group and others, these pathways are also critically involved in PXE pathogenesis and may thus explain the beneficial treatment effects of minocycline in human PXE cells, PXE zebrafish and now *Abcc6^−/−^* mice [3,12,25]. Interestingly, minocycline is also known to interfere with angiogenesis, which is an important pathomechanism in PXE retinopathy (i.e., choroidal neovascularization) [26]. Future studies may investigate whether long-term minocycline treatment also affects Bruch membrane calcification and subsequent neovascularization and hemorrhage, the latter being a major cause of decreased quality of life in PXE patients [27].

In the current study, we have increased the translational potential of minocycline towards treatment in human PXE patients by showing for the first time that minocycline is effective in reducing PXE-related ectopic calcification in a mammalian model. Several elements contribute to the favorable pharmacological and clinical profile of minocycline with regard to its possible use as an anticalcifying drug in ectopic mineralization disorders such as PXE. First, minocycline can be administered orally—with an excellent bioavailability of 95–100%—and is relatively inexpensive, which is important considering that treatment of PXE patients will potentially necessitate life-long administration of the drug since PXE is a chronic and slowly progressive disease [24]. Second, minocycline has a longer half-life and increased tissue penetration compared to first-generation tetracyclines, which may facilitate once-daily dosing regimens (200 mg/day) and increase uptake and effectiveness of the drug in peripheral soft tissues such as the skin and eyes [28]. Third, clinical experience with minocycline extends over more than 50 years and shows a good long-term safety profile with well-characterized side effects such as gastro-intestinal discomfort and dizziness (common and mild adverse effects), as well as hepatotoxicity, irreversible pigmentation and drug-induced systemic lupus erythematosus (rare but severe adverse effects) [24]. Importantly, during bone and tooth development (fetal life until 8 years of age) minocycline is contraindicated as it may result in impaired skeletal development and permanent discoloration of the teeth [29]. However, as PXE signs and symptoms typically occur from late childhood onwards (>10 years), the latter contraindication may be less relevant. Finally, alternative and novel PARP1 inhibitors, including olaparib, rucaparib, veliparib and talazoparib, are currently being developed or used for the treatment of (ovarian) cancer [30]. Additional investigations into their potential anticalcifying properties are thus warranted.

In conclusion, we validated for the first time the anticalcifying effect of minocycline, a DDR/PARP1 inhibitor, in an established murine model of PXE. Considering its favorable safety profile and low cost as a generic drug, together with the current unmet need for disease-modifying treatment options for PXE patients, future randomized and placebo-controlled clinical trials with minocycline may be envisioned.

## 4. Materials and Methods

### 4.1. Animal Studies

*Abcc6* knock-out mice were generated on a 129/Ola background and backcrossed into a C57BL/6J background more than 10 times at the Netherlands Ophthalmic Research Institute (Amsterdam, the Netherlands) as previously described [19]. These mice are designated *Abcc6^−/−^* in the current study. The animals were maintained at the Mouse Facility of the INSERM Unité Mixte de Recherche S 1155 (Paris, France) with a 12-hour dark/light cycle. All mice were fed a standard rodent diet (catalogue number 3432, Kliba Nafag, Kaiseraugst, Switzerland) and had access to drinking water ad libitum. Twelve-week-old female *Abcc6^−/−^* mice were divided into either the control group (*n* = 6) or the treatment group (*n* = 6), with the latter being treated with 200 mg/L minocycline dissolved in drinking water. Assuming a 25 g mouse drinks 5 mL of water daily, this dose corresponds to 40 mg/kg of body weight of minocycline per day. Wild-type *Abcc6^+/+^* mice (*n* = 6) received drinking water without minocycline supplementation. The treatment was continued for an additional 24 weeks and all mice were sacrificed at 36 weeks of age. The study was performed in accordance with the European Union and national guidelines on animal experimentation and Ethics Committee approval was obtained (Comité d’Éthique en Expérimentation Animale Charles Darwin, France; APAFIS #27030).

### 4.2. Histopathology and Yasue Calcium Staining of Muzzle Skin

Following euthanasia, unilateral biopsies from vibrissae-containing muzzle skin were obtained and fixed in 4% formalin for 24 h before embedding in paraffin. Four-micrometer tissue sections were cut and Yasue’s silver nitrate-rubeanic acid staining was performed to visualize calcium crystals.

### 4.3. X-ray Microtomography and Three-Dimensional Reconstruction of Vibrissae Sheath Calcification

Contralateral biopsies from vibrissae-containing muzzle skin were fixed in formalin and embedded in paraffin before imaging. X-ray computed microtomography (µCT) was performed using a Skyscan 1272 system (Bruker, Kontich, Belgium) at the Lariboisière Hospital imaging platform (Paris, France). A 6 µm resolution scale was obtained. Shadow images were obtained using an X-ray energy of 65 kV and 150 mA without filter exposition. The angular step between image acquisitions was 0.5°, and each image was averaged after 2 frames. Data were reconstructed using Nrecon software (Bruker, Kontich, Belgium) and exported into a 16-bit Tag Image File Format stack of virtual slices. The Mimics Innovation suite 20.0 (Materialise, Leuven, Belgium) was used for three-dimensional modeling and subsequent quantification analysis of vibrissae sheath calcification volume, normalized to total muzzle skin volume.

### 4.4. Immunofluorescence and Von Kossa Staining of Muzzle Skin

Immunofluorescent staining of the DDR marker H2AX was performed on paraffin sections of muzzle skin. Following deparaffinization, antigens were unmasked using 1 mM EDTA (pH 8.0, boiled for 30 min), cooled down for 1 h and subsequently immersed in 0.25% Triton X-100 in phosphate-buffered saline (PBS) for 10 min. Sections were then blocked with 5% bovine serum albumin in 0.25% Triton X-100 in PBS for 1 h at room temperature. After removal, sections were incubated with the primary antibody against H2AX (1:200, rabbit IgG, 10856-1-AP, Proteintech, Rosemont, IL, USA) for 1 h at room temperature. After washing, the secondary antibody (1:400, Alexa Fluor 488, donkey antirabbit, Cell Signaling Technology, Danvers, MA, USA) was added for 1 h at room temperature, after which slides were mounted with Vectashield containing DAPI (Vector Laboratories, Burlingame, CA, USA). To enable colocalization with calcium crystal deposits, adjacent slides were stained with 5% silver nitrate according to von Kossa and counterstained using 0.1% Nuclear Fast Red solution (Sigma-Aldrich, St. Louis, MO, USA). Digital images were then taken using brightfield and fluorescence microscopy (Carl Zeiss, Oberkochen, Germany).

### 4.5. Statistical analysis

Data are presented as mean values ± standard deviation (SD) of all measurements and compared using one-way analysis of variance (ANOVA) with Tukey’s post hoc correction for multiple comparisons. A *p* value of <0.05 was considered significant. Statistical analysis was performed using GraphPad Prism (version 9.2., GraphPad Software, San Diego, CA, USA).

## Data Availability

All data generated or analyzed during this study are included in this article.

## Supplementary Materials

The following supporting information can be downloaded at: https://www.mdpi.com/article/10.3390/ijms23031838/s1.
